# Comparative Study of Wild and Cultivated *Lavandula dentata*: Differences in Essential Oil Composition, Biological Activities, and Associated Microbial Communities

**DOI:** 10.3390/molecules30244695

**Published:** 2025-12-08

**Authors:** Siham Houssayni, Oumaima Akachoud, Btissam Zoubi, Meryem Youssfi, Anissa Lounès-Hadj Sahraoui, Frédéric Laruelle, Azucena Gonzalez Coloma, Maria Fe Andrés Yeves, Abderrazak Benkebboura, Hafida Bouamama, Ahmed Qaddoury

**Affiliations:** 1 Laboratory of Sustainable Development and Health Research, Faculty of Sciences and Techniques, Cadi Ayyad University, Marrakech 40000, Morocco; sihamhoussayni@gmail.com (S.H.); akachoud.oumaima@gmail.com (O.A.); 2 Laboratory of AgroBiotechnology and Bioengineering, Department of Biology, Faculty of Sciences and Techniques, Gueliz (FSTG) Cadi Ayyad University of Marrakech, Marrakech 40000, Morocco; btissamgcb@gmail.com (B.Z.); abderrazakbenkebboura@gmail.com (A.B.); qadahmed@gmail.com (A.Q.); 3 Unité de Chimie Environnementale et Interactions sur le Vivant, Université Littoral Côte d’Opale, UCEIV—UR n°4492, SFR Condorcet FR CNRS 3417, CEDEX CS 80699, 62228 Calais, France; anissa.lounes@univ-littoral.fr (A.L.-H.S.); frederic.laruelle@univ-littoral.fr (F.L.); 4 Instituto de Ciencias Agrarias, CONSEJO SUPERIOR DE INVESTIGACIONES CIENTIFICAS (CSIC), Serrano 115-dpdo, 28006 Madrid, Spain; azu@ica.csic.es (A.G.C.); mafay@ica.csic.es (M.F.A.Y.)

**Keywords:** *Lavandula dentata*, essential oils, gas chromatography analysis, antibacterial activity, antifungal, antioxidant activity, nematicidal properties, PLFA

## Abstract

To ensure the preservation and sustainable use of *Lavandula dentata* L., we examined the impact of various growth conditions on the composition of essential oils extracted from the leaves of both cultivated and wild *L. dentata*. Additionally, we assessed the biological activities of these essential oils, along with the biomass of the root and soil microorganisms. Gas chromatography analysis revealed 21 and 23 components in the EO of the wild and cultivated plants, accounting for over 98% of the total composition in both cases. The major compounds of wild EO were borneol (49.47%), eucalyptol (23.01%), β-pinene (3.95%), β-eudesmol (3.79%), and myrtenol (3.61%). In contrast, the EO extracted from cultivated plants was characterized by a high content of borneol (32.83%), isobornyl acetate (24.45%), eucalyptol (14.71%), and α-pinene (5.83%). Unique compounds were found in wild and cultivated EO, such as linalool, cis-verbenol, carveol, α-selinene, and terpinyl acetate or tricyclene, d-limonene, camphene hydrate, and isobornyl acetate, respectively. PLFA analysis revealed a higher microbial biomass in both soil (10.393 µg/g) and the roots (68.04 µg/g) of the wild plants compared to the cultivated ones (3.91 µg/g in soil and 62.04 µg/g in roots), driven especially by Gram-negative bacteria in soil, and by saprotrophic fungi in the roots. The biological activities of the essential oils showed some variations with growth conditions, with the wild EO generally exhibiting slightly higher antibacterial, antifungal, antioxidant, and nematicidal activities in certain assays. Overall, our findings indicate that the essential oils from wild and cultivated *L. dentata* exhibit comparable biological value, although some differences were observed. In particular, the wild EO tended to show significantly higher biological activities in certain assays, which may be associated with its distinct chemical composition and growth environment. However, these differences were moderate and not consistently significant across all tests. Therefore, properly managed cultivation can be a dependable alternative for producing *L. dentata* essential oil, helping to reduce pressure on natural populations.

## 1. Introduction

*Lavandula dentata* L. is one of the most relevant species in the Lamiaceae family, which comprises about 32 species naturally spread across the Mediterranean region, Southwest Asia, and the Arabian Peninsula [1]. In Morocco, wild populations of *L. dentata* thrive in diverse bioclimatic conditions, from the north Mediterranean coast and the High Atlas region to the Atlantic coast north of Agadir [2]. Due to its medicinal, ornamental, and melliferous benefits and properties, the species has long been valued by indigenous communities [3,4]. Nowadays, it is primarily cultivated for its essential oil (EO), which is highly sought after in the perfumery, cosmetics, and pharmaceutical industries. Among medicinal and aromatic plants (MAPs), lavender EO stands out as one of the most commercially significant due to its broad range of applications [5]. The EO of *L. dentata* is rich in bioactive compounds, including phenolic acids, flavonoids, and terpenoids, which contribute to its various biological properties [6,7,8]. Notably, its major terpenic components, linalool, camphor, eucalyptol, and linalyl acetate, are responsible for its well-documented antibacterial, antifungal, antioxidant, anti-inflammatory, and even insecticidal properties [8,9]. Additionally, *L. dentata* EO is widely used in aromatherapy for its calming and relaxing effects [10]. However, as with many MAPs, the growth, yield, and chemical composition of *L. dentata* EO are greatly affected by environmental, anthropic, and edaphic factors [11,12,13]. This fact presents a major challenge in its domestication, which is reflected in the difficulty of achieving the same bioactive properties found in wild populations [14]. Therefore, most of the commercial supply of *L. dentata* still comes from wild harvesting, often through unsustainable collection practices that threaten natural populations. Overharvesting and habitat loss have led to population declines and increased fragmentation [15]. Moreover, harvesting is typically carried out during the flowering phase before the seed set, severely limiting natural regeneration [16]. Controlled cultivation is being explored as a viable alternative, a well-established method for many medicinal plants in Europe, China, and India. However, a key challenge remains: how does cultivation influence the chemical profile and biological activity of *L. dentata* EO? Despite the widespread commercial production of lavender, there is still a need for a deeper understanding of how agricultural practices shape the quality and consistency of its EO [17]. Another crucial, yet often overlooked, factor in plant domestication is the role of microbial communities associated with *L. dentata*. The plant microbiome, specifically the microorganisms in the rhizosphere and endosphere, plays a fundamental role in plant health, nutrient uptake, and even secondary metabolite production [18,19]. Plant–microbe associations have been explored through sequencing studies, demonstrating how the EO chemical composition can be influenced by host microbial diversity [20,21,22]. However, the use of fertilizers and pesticides can significantly affect both the diversity and composition of microbial communities, leading, in consequence, to possible variations in EO chemical composition between wild, unperturbed soils and cultivated ones [23].

Given the economic and medicinal importance of *L. dentata*, understanding how cultivation affects its EO composition and biological properties is crucial. However, to our knowledge, no comprehensive studies have examined these aspects. This study aims to bridge this knowledge gap by comparing the EO chemical profile and biological activities of *L. dentata* from wild and cultivated environments. The findings will provide valuable insights into optimizing sustainable production while maintaining the plant’s therapeutic efficacy.

## 2. Results

### 2.1. Essential Oil Composition

*L. dentata* EO chemical composition analysis using Gas chromatography/mass spectrometry or GC/MS analysis permitted the identification of 21 components in wild EO and 23 in cultivated one, representing over 98% of both EO samples (Table 1). The cultivation conditions substantially affected the chemical composition of the EO samples. Overall, oxygenated monoterpenes were predominantly present in both wild (84.06%) and cultivated EO (83.68%). Regarding hydrocarbon monoterpenes, the cultivated EO exhibited a higher abundance (12.43%) compared to the wild EO (7.65%). However, hydrocarbon and oxygenated sesquiterpenes were more abundant in wild EO (1.43% and 4.85%, respectively) than in cultivated EO (0.73% and 2.54%, respectively). The main components of wild EO included borneol (49.47%), eucalyptol (23.01%), β-pinene (3.95%), β-eudesmol (3.79%), and myrtenol (3.61%). In contrast, it lacked the presence of many compounds found in cultivated EO, such as tricyclene, d-limonene, camphene hydrate, and isobornyl acetate. On the other hand, the EO extracted from cultivated plants was essentially distinguished by a higher content of borneol (32.83%), isobornyl acetate (24.45%), eucalyptol (14.71%), α-pinene (5.83%), and terpinen-4-ol (3.70%). The cultivated EO notably did not contain linalool, cis-verbanol, carveol, alpha-selinene, or terpinyl acetate, which were solely found in the EO grown in wild conditions.

### 2.2. Soil Physicochemical Properties

We collected *L. dentata* from soils exhibiting contrasting physicochemical properties between wild and cultivated conditions (Table 2). The wild soil samples collected from natural growth conditions were characterized by moderately alkaline pH. On the other hand, cultivated soil samples had lower pH values and higher acidity levels (pH water = 6.63; pH KCl = 6.13). Electrical conductivity was significantly higher in cultivated soils (1513 µs/cm) compared to wild soils (107.97 µs/cm). The organic matter content in wild soil (38.9 g/kg) was considerably higher than in cultivated one (21.67 g/kg), which aligns with the contents of organic carbon (22.60 vs. 12.60 g/kg) and nitrogen levels (3.64 vs. 2.82 g/kg). Consequently, wild soils exhibited higher carbon-to-nitrogen (C/N) ratios of 6.20, compared to 4.46 in cultivated soils. The total limestone content was significantly greater in cultivated soils (54.04 g/kg) than in wild soils (10.70 g/kg) and matched with increased calcium concentrations (17.55 vs. 11.50 g/kg). Potassium was significantly higher (1.22 g/kg) in cultivated soils than in their wild counterparts (0.53 g/kg). Sodium remained low across both soil types but was slightly more abundant in cultivated samples (0.03 g/kg) compared to wild (0.01 g/kg). Both soils were phosphorus-deficient, although cultivated soils showed slightly elevated levels (0.08 g/kg) compared to wild soils (0.03 g/kg) (Table 1).

### 2.3. Microbial Diversity in Soil and Roots

**Figure 1:**
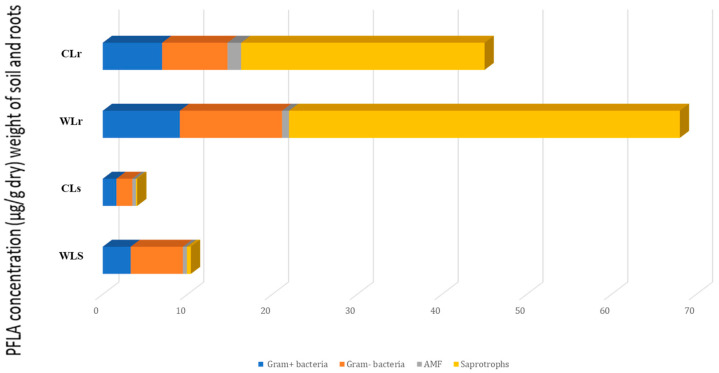
PLFA characterization of microbial communities in the roots and soil of cultivated and wild *L. dentata*, expressed in µg/g of soil or root dry weight. “CLs” refers to the cultivated *L. dentata* soil sample, and “WLs” refers to the wild soil sample collected from the Ouirgane site. “CLr” represents the cultivated root sample, while “WLr” denotes the wild root sample collected from the same commune.

Distinct disparities in total and specific microbial biomass between cultivated and wild *L. dentata* were observed using phospholipid fatty acid (PLFA) analysis (Figure 1). Both at the root and soil levels, *L. dentata* exhibited higher microbial biomass in the wild sample (WL) (10.393 mg/g in the soil and 68.04 mg/g in the roots) compared to the cultivated one (CL) (3.91 µg/g in the soil and 62.04 µg/g in the roots) (Figure 1). This disparity was especially evident in saprotrophs, the most abundant microbial group in both growth conditions, but much more abundant in *L. dentata* wild root samples (46.08 µg/g of roots) than in the cultivated root samples (22.70 µg/g of roots). Regarding Gram-positive and Gram-negative bacteria, both groups were more prevalent in the wild roots, with concentrations of 9.09 µg/g and 12.05 µg/g, respectively. On the contrary, cultivated root samples indicated lower levels at 7.00 µg/g for Gram-positive bacteria and 7.71 µg/g for Gram-negative bacteria. Arbuscular mycorrhizal fungi (AMF) were present in very low amounts in both samples, being slightly more abundant in cultivated roots (1.60 µg/g in cultivated roots, and 0.83 µg/g in wild roots). In the soil samples, the total microbial biomass was also greater in the wild soil compared to the cultivated soil. Here, Gram-positive and Gram-negative bacteria were significant contributors to this difference, with concentrations of 6.15 µg/g for Gram-positive bacteria and 3.29 µg/g for Gram-negative bacteria in wild soil, compared to 1.86 µg/g and 1.61 µg/g in cultivated soil, respectively. The Gram-negative fraction was particularly more abundant in the wild soil. Saprotrophs and AMF were present in lower amounts overall, but their levels were still slightly higher in the wild soil compared to the cultivated soil, at 0.47 µg/g and 0.42 µg/g for AMF, and 0.46 µg/g and 0.14 µg/g for saprotrophs, respectively.

### 2.4. Biological Activities

#### 2.4.1. Nematicidal Activity

**Figure 2:**
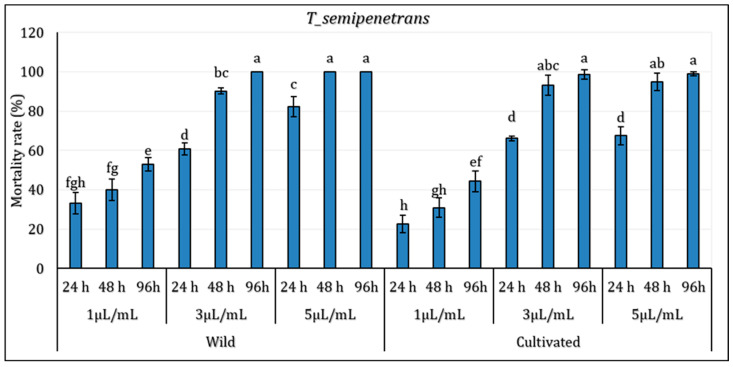
Mortality (%) of *Tylenchulus semipenetrans* juveniles exposed to essential oils from Wild (WL) and Cultivated (CL) *L. dentata* at different concentrations and exposure times. The values are presented as means (*n* = 5) with standard deviation bars. Different letters above the bars indicate statistically significant differences between treatments according to Student’s *t*-test (*p* < 0.05).

The results indicated that mortality was significantly influenced by both concentration and exposure time, with some variations observed between wild (WL) and cultivated (CL) essential oils (*p* ≤ 0.05) (Figure 2). The EO extracted from wild (WL) and cultivated (CL) *L. dentata* plants showed notable nematicidal activity against the pathogenic nematode *T. semipenetrans* (j2). At the lowest concentration (1 µL/mL), a moderate mortality rate of *T. semipenetrans* juveniles was observed following a 24 h exposure to both wild (WL) and cultivated (CL) *L. dentata*’s EO (33.11% and 22.74% for wild and cultivated EO, respectively). In particular, mortality at 1 µL/mL remained moderate even after 96 h, implying that the nematicidal activity depends on concentration. With an intermediary effect between the lowest and highest concentrations, the response to 3 µL/mL also followed a similar pattern. At higher concentrations and longer exposure times, mortality rates did rise noticeably. With the highest values noted at 5 µL/mL, WL’s mortality rate was higher than in CL at all concentrations at 48 h. After 96 h of exposure to WL, *T. semipenetrans* j2 mortality reached 100% at 5 µL/mL; in CL, it exceeded 98%. Although WL still showed somewhat higher nematicidal activity, by 96 h, the difference between WL and CL became less noticeable.

**Figure 3:**
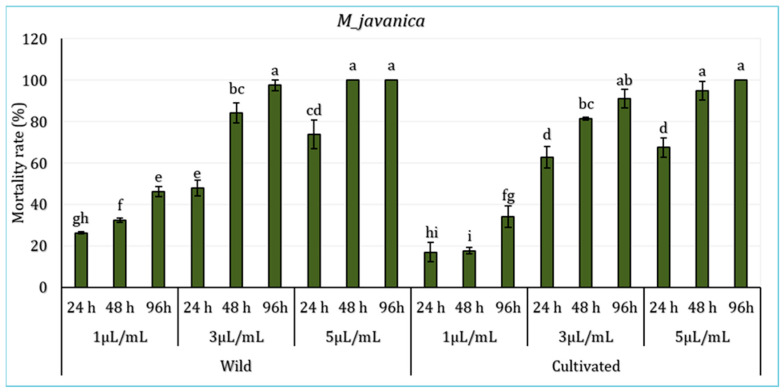
Mortality (%) of *Meloidogyne javanica* juveniles exposed to essential oils from Wild (WL) and Cultivated (CL) *L. dentata* at different concentrations and exposure times. The values are presented as means (*n* = 5) with standard deviation bars. Different letters above the bars indicate statistically significant differences between treatments according to Student’s *t*-test (*p* < 0.05).

The effect of *L. dentata*’s EO in various concentrations (1 μL/mL, 3 μL/mL, and 5 μL/mL) (Figure 3) on the juveniles of the borne pathogen nematode *M. javanica* during 94 h was evaluated. Statistical analysis revealed significant differences among periods of exposure and treatments, particularly between the lower and higher concentrations (*p* < 0.05), with no significant variations registered between the sites (*p* > 0.05). Results showed that the mortality rate varies depending on oil concentration, exposure time, and plant origin (wild or cultivated). In wild *L. dentata*’s EO samples, the 5 μL/mL treatment showed the highest mortality rate, reaching 100% after 96 h. The treatment with a 3 μL/mL concentration demonstrated equally significant nematicidal activity, with a mortality rate exceeding 97% at 96 h. In contrast, the lower concentration of 1 μL/mL induced the lowest mortality, reaching around 46% by the end of 96 h. Likewise, the cultivated *L. dentata* (CL) demonstrated a concentration and time-dependent effect, which was lower than wild EO samples. The highest concentration (5 μL/mL) induced 100% mortality at 96 h, while the 3 μL/mL treatment ranged around 91%. The lowest mortality was registered with the lowest treatment (1 μL/mL), with values between 17% and 34% depending on the exposure period.

#### 2.4.2. Anti-Fusarium Activity

**Figure 4:**
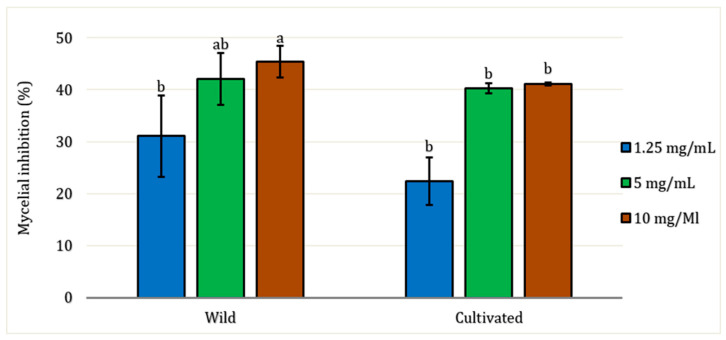
Inhibition of radial growth of *Fusarium oxysporum* by aerial part essential oil of *L. dentata* at different concentrations (mg/mL). The values are presented as means (*n* = 3) with standard deviation bars. Different letters above the bars indicate statistically significant differences between treatments according to Student’s *t*-test (*p* < 0.05).

All EO samples demonstrated antifungal activity against the pathogenic fungi *Fusarium oxysporum* at all concentrations tested (Figure 4). There were no significant differences (*p* > 0.05) based on the factors of site and concentration, except for the 10 mg/mL concentration, where variation in mycelial inhibition was observed between wild and cultivated EO (*p* < 0.05). The lowest concentration tested (1.25 mg/mL) exhibited mycelial inhibition against the fungal strain of only 31% for wild EO and 22.40% for cultivated EO. The maximum concentration tested was 10 mg/mL, which showed the highest inhibition rates for both EO: 45.38% for wild and 41.12% for cultivated, respectively.

#### 2.4.3. Antibacterial Activity

**Figure 5:**
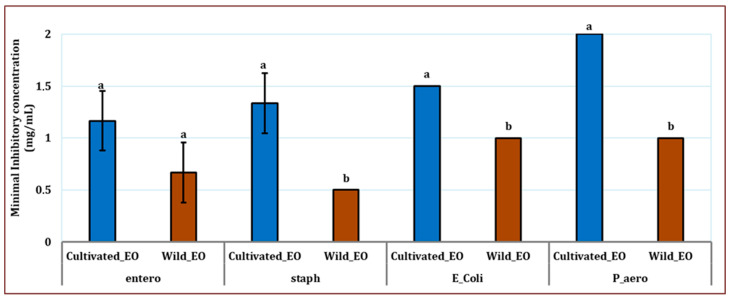
Minimal Inhibitory Concentration (in mg/mL) of Wild and Cultivated *L. dentata* essential oil against Four Bacterial Strains: *Escherichia coli* (E_Coli), *Staphylococcus aureus* (staph), *Pseudomonas aeruginosa* (P_aero), and *Enterococcus hirae* (entero). The values are presented as means (*n* = 3) with standard deviation bars. Different letters above the bars indicate statistically significant differences between treatments according to Student’s *t*-test (*p* < 0.05).

The results reported in Figure 5 show the antibacterial assays of *L. dentata* EO against four selected microorganisms representative of the Gram-positive and Gram-negative bacteria. All samples (wild and cultivated EO) exhibited antibacterial activity against all tested microorganisms.

The antibacterial activity of EO against all bacterial strains varied significantly according to the growth conditions (*p* ≤ 0.05) except for *E. hirae* (*p* ≥ 0.05) (Figure 5). MIC values indicated that the Gram-positive bacterial strains were generally the most sensitive among the microorganisms tested. MIC values ranged from 0.5 mg/mL to 1.33 mg/mL for wild and cultivated EO. The MIC values ranged from 1 mg/mL to 2 mg/mL for Gram-negative bacterial strains. Overall, the EO extracted from natural growth conditions generally exhibited higher antibacterial activity than plants grown in the cultivated site against all tested bacterial strains. The lowest MIC registered against *E. hirae* was observed for the wild EO (0.66 mg/mL), while the cultivated oil was less effective (1.16 mg/mL). For *S. aureus*, the pattern remained similar; the value for the wild EO was 0.5 mg/mL, whereas 1.33 mg/mL was recorded for the cultivated oil. Regarding *E. coli*, the wild oil was again more active (1.00 mg/mL), while the cultivated oil was less effective (1.50 mg/mL). *P. aeruginosa* was the most resistant strain among those tested, with the wild EO showing inhibition at 1 mg/mL. In contrast, the cultivated oil exhibited the weakest activity, with 2.00 mg/mL as a MIC.

#### 2.4.4. Antioxidant Activity

**Figure 6:**
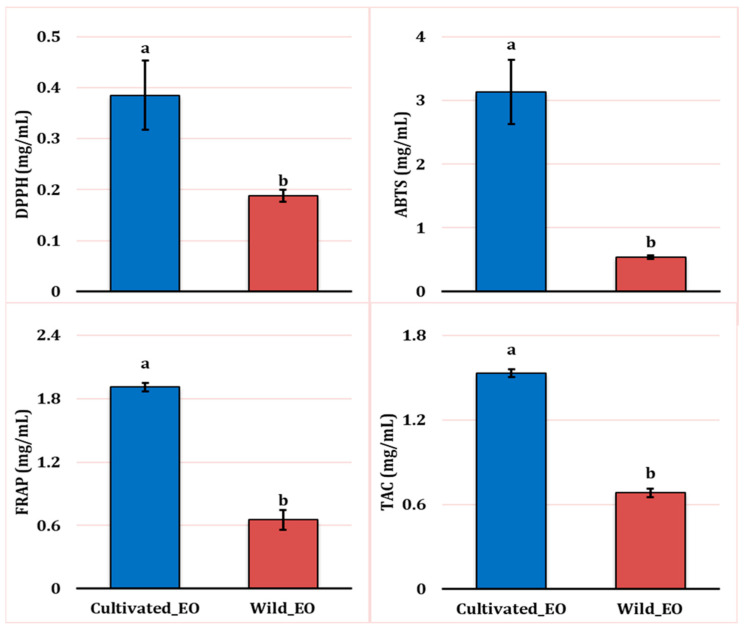
Antioxidant activities of wild and cultivated *L. dentata* essential oils based on DPPH, FRAP, ABTS, and TAC assays (mg/mL). The values are presented as means (*n* = 3) with standard deviation bars. Different letters above the bars indicate statistically significant differences between treatments according to Student’s *t*-test (*p* < 0.05).

All EOs obtained from wild and cultivated plants exhibited antioxidant activity (Figure 6). The results of the four antioxidant assays (ABTS, DPPH, TAC, and FRAP) showed significant differences (*p* ≤ 0.05) between the oils, with the cultivated *L. dentata*’s EO displaying lower activity than the wild oil across all tests.

The DPPH assay revealed weaker radical scavenging activity in the cultivated EO (0.38 mg/mL) compared with the wild oil samples (0.18 mg/mL). Similarly, we observed a higher reducing potential in the wild oil (0.65 mg/mL) compared to the cultivated one (1.91 mg/mL) using the FRAP assay. Likewise, the total antioxidant activity method (TAC) showed that EO extracted from wild plants had higher activity (0.68 mg/mL) than cultivated EO (1.53 mg/mL). Lastly, following the same pattern, the ABTS assay indicated weaker antioxidant activity in cultivated oil at 3.13 mg/mL, compared to wild EO at 0.54 mg/mL.

### 2.5. Essential Oil Yield (%) of Cultivated and Wild Lavandula dentata

**Figure 7:**
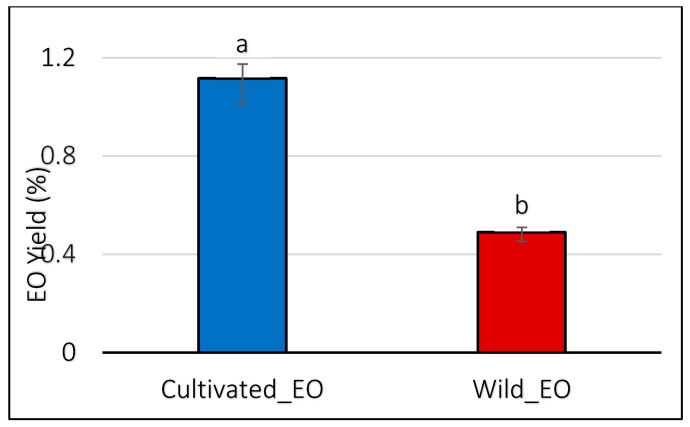
Essential oil yield between cultivated and wild *L. dentata*. The values are presented as means (*n* = 3) with standard deviation bars. Different letters above the bars indicate statistically significant differences between treatments according to Student’s *t*-test (*p* < 0.05).

The results in Figure 7 show that the essential oil yield of cultivated *L. dentata* (1.11%) was significantly higher than that of wild plants (0.49%) (*p* < 0.05).

### 2.6. Heatmap Correlation Analysis

**Figure 8:**
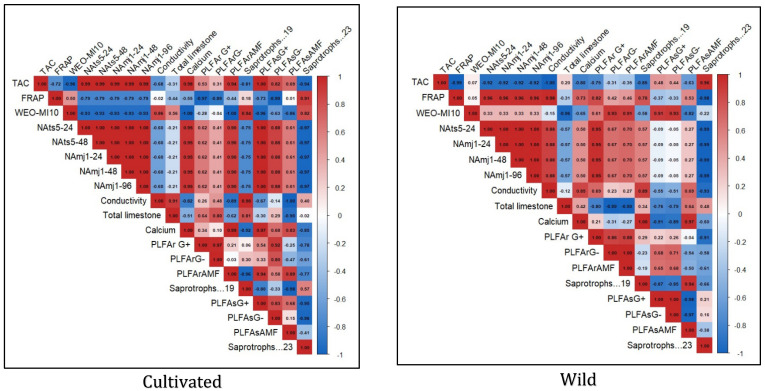
The heatmap illustrates the correlation between microbial abundance, biological activities, and physicochemical parameters of soil in wild and cultivated *L. dentata* samples. The data sets include root and soil compartments, with the values expressed as micrograms per gram dry weight. The coloration gradient indicates the strength and directionality of the correlations: strong positive correlations in red shades, while strong and moderate negative correlations in shades of blue. PLFAsAMF (AMF concentration in soil); PLFASG (Gram-negative bacteria concentration in soil); PLFArAMF (AMF concentration in roots); PLFArG (Gram-negative bacteria concentration in roots); PLFArG (Gram-positive bacteria in roots); NAmj (nematicidal activity against *Meloidogyne javanica*); NAts (nematicidal activity against *Tylenchulus semipenetrans*); WEO.MI10 (mycelial inhibition of *Fusarium oxysporum* activity).

The Heatmap correlation analysis clearly shows a divergence in the ecological interactions of cultivated and natural *L. dentata* ecosystems (Figure 8). The wild soil sample shows a strong positive correlation between TAC and saprotrophic groups (r = 0.96), while it was negatively correlated with PLFAsAMF (r = −0.63). FRAP exhibited a very strong positive correlation with soil conductivity (r = 0.98), a moderate positive correlation with calcium (r = 0.73), and a weak negative correlation with total limestone (r = −0.31). FRAP also showed a moderate positive correlation with PLFAsAMF (r = 0.53). Regarding nematicidal activity, it presented a very strong negative correlation with saprotrophs (r = −0.99), a weak positive correlation with AMF diversity (r = 0.27), and a negligible negative correlation with Gram-negative bacteria in soil (r = −0.05), while it exhibited a positive correlation with bacterial diversity in roots (r = 0.67 to 0.95). In contrast, the cultivated samples exhibited weaker and more fragmented patterns. TAC showed negative correlations with several soil properties, including electrical conductivity (r = −0.68) and total limestone content (r = −0.31). Additionally, TAC had a weak correlation with saprotrophs, ranging from r = −0.81 to −0.94. FRAP, on the other hand, demonstrated a strong positive correlation with saprotrophs (r = 0.91) but negative correlations with most other parameters. Nematicidal activity showed moderate positive correlations with Gram-positive (r = 0.62) and Gram-negative (r = 0.41) bacteria in roots, and strong correlations with the same bacterial groups in soil (r = 1.00 and r = 0.88, respectively).

## 3. Discussion

Medicinal and aromatic plants are prominent in promoting human development and welfare, and they hold major cultural value among many indigenous communities both within and outside Morocco [24,25]. These herbs synthesize many secondary metabolites, such as terpenes and phenolic compounds, which endow them with many biological activities (antibacterial, antifungal, and herbicide) [26]. Conversely, the wild species in Morocco are undergoing a very significant degradation and decline due to the intense exploitation and overuse of these plants for medical and aromatic needs and purposes. Most medicinal and aromatic species used in Morocco come from wild sources; therefore, cultivating these plants is becoming a dependable solution to reduce the overgrowing pressure on wild aromatic and medicinal plants and to ensure a stable supply of EO with desirable chemical and biological properties. This study revealed distinct differences between wild and cultivated *Lavandula dentata* L. in microbial diversity, chemical profile, and biological activities, all influenced by environmental and cultivation conditions. Chemical analysis of wild and cultivated *L. dentata* EO identified 21 and 23 compounds in wild and cultivated EO, representing 98% and 99.39% of the total oils, respectively. Oxygenated monoterpenes (84.06–83.68%) were the dominant subclass, with Borneol (49.47–32.83%) as the major compound, followed by Eucalyptol (23.01–14.71%) and Terpinen-4-ol (3.10–3.70%). These compounds are commonly found in various Moroccan and Mediterranean *L. dentata* EO, but their relative contents differ based on geographic origin, growth conditions, and environmental factors [27,28,29]. Our findings align with other studies in which Borneol and Eucalyptol were the major components of this species [8,9,10,11,12,13,14,15,16,17,18,19,20,21,22,23,24,25,26,27,28,29]. Although other studies found that eucalyptol and borneol were recorded in lower amounts, there were some discrepancies in the findings compared to other regions of Morocco, where camphor and linalool were identified as the main compounds or chemotypes [13,14,15,16,17,18,19,20,21,22,23,24,25,26,27,28].

Our results demonstrated differences between essential oils obtained from cultivated and wild accessions. For instance, both plant origins exhibited unique components present in one and absent in the other. Wild EO revealed the presence of linalool, cis-verbenol, carveol, alpha-selinene, and terpinyl acetate. In contrast, other distinct compounds, including tricyclene, d-limonene, camphene hydrate, and isobornyl acetate, distinguished cultivated EO. Quantitative and qualitative differences noted in the chemical profile of *L. dentata* between different growth conditions were reported in previous studies for other aromatic and medicinal plants such as *Thymus pallidus*, *Thymus satureioides*, *Glechoma hederacea*, and *Foeniculum vulgare* [16,17,18,19,20,21,22,23,24,25,26,27,28,29,30,31]. These chemical changes may be attributed to environmental factors such as nutrient availability and microbial interactions, which play a crucial role in shaping the chemical composition of secondary metabolites. Among these factors, soil is one of the most influential, as it regulates air, nutrients, and water availability, directly impacting plant metabolism [32]. Soil nutrients such as nitrogen, phosphorus, potassium, and microelements are essential for metabolic and structural processes, including enzyme activity, photosynthesis, and the biosynthetic pathways of secondary metabolites [33,34]. A deficiency in these elements can lead to metabolic shifts, altering the production of EO key compounds. Our study revealed significant differences in the physicochemical properties of wild and cultivated soils, which may explain the observed variations in EO composition. Wild *L. dentata* soil exhibited a slightly alkaline pH, higher organic matter content, and greater nitrogen levels, whereas cultivated soil contained higher calcium, potassium, sodium, and phosphorus concentrations. These disparities reflect distinct soil formation processes and the influence of agricultural practices, which modify soil composition and structure, ultimately affecting plant metabolism and secondary metabolite synthesis.

Agricultural practices, particularly fertilization, can increase phosphorus and potassium availability, as seen in the cultivated soil [35,36]. However, phosphorus plays a crucial role in terpenoid biosynthesis, as it is involved in the production of isopentenyl diphosphate (IPP) and dimethylallyl pyrophosphate (DMAPP), precursors in the synthesis of monoterpenes and sesquiterpenes [37]. While phosphorus is essential for terpenoid production, excess levels can shift metabolic pathways and produce quantitative and qualitative differences in EO composition. For instance, in a study on *Lavandula intermedia*, phosphorus-increased application led to a decrease in linalool content from 41.82% to 38.54% [38]. These findings suggest that phosphorus influences the biosynthetic pathway of terpenoids, leading to quantitative and qualitative changes in EO composition. It could partly explain why cultivated *L. dentata* EO maintains a similar oxygenated monoterpene content to its wild counterpart but has lower hydrocarbon and oxygenated sesquiterpenes.

Soil analysis showed that the wild site contained higher organic matter compared to the cultivated site. Indeed, several studies confirmed our results. For instance, a study investigating the variation in soil organic carbon related to land use changes showed that, when comparing different land use types and all soil depths, the overall average soil organic carbon stock was higher under natural and mixed forest land use [39]. Similarly, another study revealed significant differences between organic matter content in natural ecosystems, such as forestland (3.24%), compared to cultivated large-scale conventional farming of fruit orchards (1.84%) [40].

Organic matter is crucial in nutrient cycling and soil fertility, directly influencing plant growth and metabolism [41]. Hoffland et al. explain that wild soils, being largely undisturbed, allow for the natural accumulation of organic matter through processes such as plant root exudation and litter decomposition [42]. This accumulation enhances soil structure, moisture retention, and nutrient availability, all of which can contribute to differences in secondary metabolite production between wild and cultivated *L. dentata*. For instance, a study on *Crocus sativus* demonstrated that soils abundant in organic matter significantly increased concentrations of specific secondary metabolites, such as trans-4-GG crocins, while decreasing safranal levels [43].

Furthermore, nitrogen, which was also more abundant in wild soil, is another key element known to enhance terpenoid production by increasing photosynthetic activity and providing the carbon substrates necessary for isoprene synthesis [44,45]. The higher nitrogen availability in wild soil could contribute to the increased hydrocarbon and oxygenated sesquiterpenes observed in wild EO. Indeed, a study conducted on *Z. clinopodioides* revealed that nitrogen played an important role in activating enzymes involved in the production and metabolism of EO, particularly in the biosynthesis and accumulation of monoterpenes [46,47]. These studies further confirm the role of soil nutrients in the variation in EO chemical composition. Thus, the differences in soil composition between wild and cultivated *L. dentata* reflect broader metabolic shifts in the plants, resulting in variations in EO quality.

Phospholipid fatty acid analysis showed that the microbial biomass of roots was greater than that of the rhizospheric soil at both cultivated and wild sites. This observation is in agreement with other reports that roots provide a favorable microenvironment for microbe colonization, mostly because of the exudation of root products rich in sugars, amino acids, and organic acids that serve as sources of nutrients for the microbiome [48,49,50]. Thus, these exudates favor the increase in the amount and density of the microbes in the root zone. Furthermore, microorganisms inhabiting the endosphere experience less competition and environmental stress than those living in the soil, which supports their thriving population [51]. It is also worth noting that PLFA analysis particularly detects active microbial populations, where microbial viability is more pronounced [52].

PLFA analysis further revealed that the roots and soil of wild *L. dentata* harbored a higher abundance of microorganisms, including Gram-positive, Gram-negative bacteria, and saprotrophic fungi. The variation in microbial biomass between both sites is positively correlated with the difference in organic matter, as many studies in this context were conducted where a direct correlation between organic matter and soil microbiota biomass was observed [53,54,55]. Organic matter can be easily decomposable by bacteria [56], which can also explain the high observed biomass of bacteria, regardless of negative or positive gram bacteria in the soil, compared to the fungi biomass. Furthermore, this difference may be explained by the fact that the over-application of inorganic fertilizers can lead to soil degradation. While they provide plants with nutrients, they can also alter soil composition by changing microbial communities. Several studies focused on the negative effects of cultivation practices on microbial communities, particularly bacterial biodiversity [57,58]. In this context, it has been revealed that the bacterial community of both Gram-positive and Gram-negative bacteria decreased following long-term fertilizer application [59]. This decrease may lead to changes in soil pH due to the continuous application of chemical fertilizers, resulting in a shift in bacterial community size and structure, which aligns with our results. Furthermore, fertilizer application significantly changed the bacterial community’s diversity, abundance, and composition in *Zea mays*, particularly when applied at high levels. Redundancy analysis showed that the main factors influencing these changes were a drop in pH and an increase in available nutrients brought on by fertilizer application [60]. Similarly, in a study conducted on ginseng, the authors found a higher bacterial diversity in the rhizosphere of wild ginseng in comparison with its cultivated counterpart [61].

A study on mycorrhizal responses in crop and wild plants revealed that plants growing in natural conditions can sometimes form negative associations that can limit fungal interactions. Furthermore, higher nutrient availability in cultivated systems may further enhance mycorrhizal proliferation and association, whereas, in wild conditions, AM fungi may face more competition for limited resources [62]. Moreover, a study on wild tomatoes and oats revealed lower mycorrhizal dependency, which has been linked to their lower inherent growth rates and reduced nutrient demand as adaptations to the limited nutrient availability in wild growth conditions [63]. It explains the higher abundance of mycorrhizal fungi in cultivated roots compared to the natural ecosystem. In contrast, other studies have revealed findings that contradict our results, indicating that the colonization of mycorrhizal fungi species in cultivated plants decreases with agricultural intensification, which is characterized by high tillage frequency, low crop diversity, and high nutrient input [64,65,66].

The observed differences in microbial diversity between natural and cultivated conditions suggest that microbial diversity and abundance may play a major role in the variation in the composition and quantity of *L. dentata* EO. Indeed, based on recent advances in microbial disciplines, some documented plant resistance mechanisms, such as the production of specific secondary metabolites, may be attributed to endophytes, which are obscure organisms that reside within the tissues of plants [67]. According to Zhai et al., endophytes stimulate secondary metabolite accumulation via plant defense mechanisms, triggering biosynthetic pathways that lead to increased production of secondary metabolites such as terpenes [68]. For instance, *Pseudomonas fluorescens*, an endophytic bacterial strain isolated from *Atractylodes lancea*, significantly enhanced sesquiterpenoid accumulation in its host plant compared to the control, probably via the activation of the mevalonic acid pathway, leading to the synthesis of isopentenyl pyrophosphate, the key precursor of terpenoid biosynthesis [69]. Moreover, *Penicillium oxalicum* has been reported to enhance artemisinin accumulation in *Artemisia annua* [70]. Endophytes isolated from *Atractylodes lancea* have been shown to increase sesquiterpenoid accumulation in host plants without affecting plant biomass [69,71]. This fact suggests that rather than increasing plant biomass, *A. lancea* preferentially uses the endophyte-increased photosynthate to produce secondary metabolites. Rhizospheric microorganisms are also known for their potential to produce volatile organic compounds (VOCs), mostly carbon-containing substances, including terpenes, alcohols, and ketones [72]. Moreover, some studies focused on microorganisms’ capacity to perform biotransformation. For instance, one study by Kashid et al. investigated the capacity of *Rhodococcus erythropolis* and *Pseudomonas putida* to transform the chemical composition of d-limonene through biotransformation. The results showed that R. erythropolis converted d-limonene into l-carveol while *P. putida* transformed it into S-(−)-perillyl alcohol and S-(−)-perillic acid [73].

The variation in the chemical profile of EO from one site to another affected its biological properties. Our results revealed moderate differences in biological activities, including antibacterial, antioxidant, and nematicidal properties, between wild and cultivated *L. dentata*, although these differences were not consistent across all assays. Concerning EO antibacterial activities, the results showed the effectiveness of wild EO compared with cultivated ones against the tested bacterial strains. Moreover, EOs from both sites were more active against Gram-positive than Gram-negative bacteria. Due to the additional defense provided by the outer membrane, which contains lipopolysaccharide, Gram-negative bacteria are typically more resistant to antimicrobial treatments than Gram-positive bacteria. [74]. The significant antibacterial properties in both *L. dentata* samples could be due to the high content of oxygenated monoterpenes and sesquiterpenes, including borneol, eucalyptol, and camphor. The presence of borneol, a major constituent of EO extracted from various medicinal and aromatic plants, has been linked in numerous studies to the antimicrobial qualities of various EO [75,76,77]. Borneol’s mechanism of action against microbial cells is by altering their permeability and integrity, which leads to leakage of ions and metabolites [78]. In addition, owing to the hydrophobic properties of borneol, it can reduce bacterial adhesion by forming a physical barrier via a membrane disruption mechanism [79].

The antibacterial activity of 1,8-cineole, also known as eucalyptol, has been demonstrated against several Gram-positive pathogenic strains, including *Streptococcus aureus*, *Bacillus subtilis*, *Streptococcus pyogenes*, *Bacillus cereus*, and *S. aureus*, as well as Gram-negative strains, including *E. coli*, *P. aeruginosa*, *Salmonella enteritidis*, and *Salmonella typhimurium* [80,81,82]. Studies have shown that administering 1,8-cineole caused the downregulation of genes linked to membrane proteins and carbohydrate metabolism at the mRNA level [83]. Camphor, an oxygenated monoterpene, is found in the EO of numerous medicinal and aromatic plants, including *L. dentata*, and has shown potency against multiple pathogens, including *S. aureus* and *E. coli*. Camphor disrupts bacterial cell membranes, induces apoptosis, and interferes with their metabolic pathways [84]. Both wild and cultivated plants’ EO contain these compounds. The wild EO sample showed the strongest antibacterial activity, indicating that its shared components alone do not account for the observed effects. The biological properties of other minor components, such as carveol, camphene, cis-verbenol, and linalool, have also been documented [20,85,86]. The differences in antioxidant activity between the EO of wild and cultivated *L. dentata* can be attributed to the qualitative and quantitative variations primarily caused by natural growing conditions. These conditions lead to a higher content of oxygenated sesquiterpenes and monoterpenes, which are recognized for their strong antioxidant properties [87,88,89].

The tested lavender EO from both growth conditions demonstrated high mortality rates against the root-knot nematode *M. incognita* and the semi-endoparasitic nematode *T. semipenetrans*. In line with our results, several studies confirm the nematicidal activity of Lavandula EO against several species of nematode-borne pathogens [90,91,92]. Overall, the nematicidal activity of EO is governed by its major chemical components. Therefore, the toxicity of *L. dentata* is mainly linked to the high content of oxygenated monoterpenes, which are well-documented for their nematicidal effects [91,92,93]. The molecular structure and its functional groups have been shown to influence its toxic effects on nematodes. Generally, a higher degree of oxygenation and unsaturation enhances nematicidal activity [91]. Both EOs contain a high percentage of oxygenated molecules (84.06% and 83.68%), including linalool, camphor, and eucalyptol. According to Dutta et al., these compounds exert nematicidal effects through various mechanisms, depending on the type of molecules involved. Often, these components work together synergistically for a stronger effect [94]. Due primarily to their lipophilic characteristics, terpenes primarily inhibit nematodes by altering the permeability of the plasmic membrane. This disruption compromises the membrane’s barrier function, leading to leakage of cytoplasmic macromolecules and ultimately causing cell death in phytoparasite nematodes [95].

Regarding the antifusarium activity of the essential oils, the wild *L. dentata* sample generally exhibited higher inhibitory effects than the cultivated sample across the tested concentrations, although the differences were not always statistically significant. The differences in bioactivity observed between EO from the two growth conditions may be attributed to variations in the interaction between their structural components [96]. Although the major constituents have a predominant role, the presence and ratios of minor compounds could also contribute through synergistic effects and modulate overall biological activity. Of these, linalool is the most well-documented and has strong biocidal effects against phytopathogenic fungi and clinical fungal strains. Zore et al. reported the antifungal activity of linalool against 39 clinical strains of *Candida albicans*, indicating that linalool is a broad-spectrum fungicidal agent [97]. Similarly, (+)-β-pinene has demonstrated significant antifungal effects, with minimum inhibitory concentrations (MICs) ranging from 56.25 to 1800 µmol/L against *Candida* spp., as reported by de Macêdo Andrade et al. [98]. The study further described its antifungal mode of action, anti-biofilm properties, and potential for membrane interaction, suggesting comparable mechanisms may be involved in the antifungal effects observed in our *L. dentata* EO.

The Heatmap correlation analysis clearly shows a divergence in the ecological interactions of cultivated and natural L. dentata ecosystems. In the wild soil sample, the strong positive correlation between TAC and saprotrophic fungi is likely reflecting the enhanced organic matter decomposition and nutrient mineralization by saprotrophs [99], which in turn stimulates phenolic and antioxidant metabolism in the plant [100,101,102]. Similarly, the very strong positive correlation between FRAP and soil conductivity and Ca^2+^ suggests that higher ionic composition and Ca^2+^ availability promote antioxidant activity through stress signaling and phenolic biosynthesis pathways [103,104].

The strong negative correlation between nematicidal activity and saprotrophs suggests that when decomposition processes dominate, fewer resources are directed toward defense metabolite production, reducing nematode suppression [105]. The weak positive correlation with AMF diversity indicates that mycorrhizal associations may modestly enhance nematicidal potential through systemic defense priming [106,107]. In contrast, the positive correlation with root bacterial diversity highlights the key role of bacteria in producing nematicidal compounds and inducing plant resistance [108].

In contrast, the cultivated samples exhibited weaker and more fragmented patterns. This suggests that cultivation practices may alter carbon cycling and microbial community structure, reducing the influence of decomposers on plant secondary metabolism [109]. Nematicidal activity showed moderate positive correlations with Gram-positive and Gram-negative bacteria in roots, and strong correlations with the same bacterial groups in soil, which further indicate that these bacterial groups likely act synergistically within the rhizosphere, enhancing biological control potential through competitive interactions and secondary metabolite secretion [110].

## 4. Materials and Methods

### 4.1. Study Site and Sampling

In February 2024, wild and cultivated *L. dentata* samples, including aerial parts, roots, and soil, were collected during the flowering stage of development from the rural commune of the Ouirgane region in Marrakech-Safi. The wild plants were collected at the coordinates 31°10′02.5″ N 8°02′26.0″ W, while the cultivated *L. dentata* was obtained from a small, self-managed cultivation site located at 31°10′24″ N 8°04′30″ W. Cultivated plants came from vegetatively propagated cuttings taken from the local wild population, ensuring a shared genetic background between wild and cultivated groups. The taxonomic identification of the plant material was done by Professor Ahmed Ouhammou, a faculty member in the Biology Department at the Faculty of Sciences Semlalia of Marrakech (FSSM), Morocco, affiliated with the Biotechnologies, Protection, and Valorization Laboratory of Plant Resources at FSSM.

Soil and root samples were collected using a randomized method. We have cleared about twenty to forty centimeters around the roots for the soil samples. To create a composite sample, we have taken approximately 5 g of fine roots and 2 kg of rhizospheric soil from different locations around the five plants. Stems and leaves were cut clean with scissors and placed in sealed bags, while the roots were pulled gently to prevent damage and stored in sterile bags. For physico-chemical and biological testing, we kept the root and soil samples at room temperature, 28 °C and −20 °C, respectively. Plant material was collected from 10 randomly chosen *L. dentata* individuals at each site (wild and cultivated). The aerial parts from each plant were combined to create a composite sample representing each population, which was then used for essential oil extraction. The plant material was dried in a shaded area at room temperature before being used for chemical and biological analyses.

### 4.2. Soil Physicochemical Properties Analysis

All physicochemical analyses of the soil samples were conducted using dried and sieved 2 mm samples, per standardized procedures. Following Richard et al. [111] approach, we measured the pH and electrical conductivity (EC) in a 1:2.5 (*w*/*v*) soil: water suspension or soil: KCl (1 M) solution to measure pHKCl. Organic carbon and matter content were assessed using Anne’s method, as detailed by Aubert [112]. The Kjeldahl method, modified by Barbano [113], was used to determine the total nitrogen concentration. Available phosphorus was obtained using the extraction method of Olsen et al. [114] based on the extraction of orthophosphates with a sodium dichromate solution. We determined the total limestone content using Bernard’s calcimeter method, which measures CO_2_ release upon treatment with hydrochloric acid (HCl), as Michel-Dewez et al. [115] described. The concentrations of potassium (K^+^), sodium (Na^+^), and calcium (Ca^2+^) were measured using flame photometry, according to the procedure established by Brown and Lilleland [116].

### 4.3. Essential Oils Extraction and GC-MS Analysis

#### 4.3.1. Hydrodistillation Process

Forty grams of lavender’s dried leaves were subjected to hydrodistillation for 3 h using a Clevenger apparatus. The extraction procedures were carried out in triplicate, and the obtained EO was recovered and then kept in a refrigerator in hermetically closed opaque-glass flasks at 4 °C.

#### 4.3.2. Essential Oils Chemical Composition

EO samples were mixed with ethyl acetate in a 1:200 *v*/*v* ratio and analyzed using an Ultra gas chromatography–mass spectrometer (GC-MS) Shimadzu QP-2021 (Shimadzu, Kyoto, Japan). The system features a single quadrupole mass spectrometer (GC-MS) and a flame ionization detector (FID). Helium was utilized as the carrier gas, maintaining a constant linear velocity of 60 cm/s. A volume of 0.2 μL of the pre-diluted EO sample was injected with a 1:10 split ratio at 260 °C into a ZB-5MS capillary column (Phenomenex, Le Pecq, France). The column, measuring 10 m in length with a 0.10 mm inner diameter and a 0.10 μm phase thickness, comprised 5% phenylacetylene and 95% dimethylpolysiloxane. The temperature program started at 60 °C (held for 2 min) and increased linearly to 280 °C (held for 1 min) at 40 °C/min. Mass spectra were recorded with an ionization energy of 70 eV and an interface temperature of 280 °C, covering a mass range of 35–350 *m*/*z*.

EO compounds were later identified by comparing their Kovats indices, obtained from retention times, with those reported in the literature, the pherobase, and the NIST database after injecting n-alkanes under the same conditions. The identification of the chemical compounds was further confirmed by matching the obtained mass spectra with those in the National Institute of Standards and Technology, Gaithersburg, MD, USA database. The relative percentage of EO compounds was determined by calculating the area under each chromatographic peak.

### 4.4. Analysis of Soil Fatty Acids

According to the Frostegård method, 3 g of lyophilized soils and roots were added to chloroform, methanol, and citrate buffer (0.5:1:0.4 *v*:*v*:*v*). The mixture was then stirred vigorously using a vortex mixer and left at room temperature for two hours. After this period, it was centrifuged, and the lipid-containing phase was collected and evaporated under nitrogen. A C19 internal standard (0.25 µg/mL) was added to the samples, which were evaporated under nitrogen at 40 °C. Subsequently, we conducted the lipid class separation using SPE-Silica columns (6 mL volume, 500 mg sorbent, Interchim, Montluçon, France), and neutral lipids (NLFA) were eluted with chloroform, glycolipids with acetone (which was discarded), and phospholipids (PLFA) with methanol. The NLFA and PLFA fractions were dried under a stream of nitrogen. Following this, we conducted transesterification using a toluene/methanol mixture at a 1:1 ratio, and KOH (0.2 M in methanol). To finalize the process, we performed an extraction using a 4:1 hexane/chloroform mixture, as well as 1 M acetic acid and water. The upper phase containing free fatty acid methyl esters (FAMEs) was collected, evaporated under nitrogen, and stored at −20 °C. For GC-MS analysis, samples were reconstituted in hexane, transferred to GC vials, and analyzed using a gas chromatography–mass spectrometer (GC-MS) Shimadzu QP-2010 Ultra (Shimadzu, Kyoto, Japan) equipped with a single quadrupole mass spectrometer detector (MS) and simultaneously coupled with a flame ionization detector (FID). Samples were analyzed in split mode (80:1 ratio) on a ZB-1MS fast capillary column (10 m length × 0.1 mm inner diameter × 0.1 μm phase thickness, 100% dimethylpolysiloxane, Zebron, Phenomenex, Torrance Calif, CA, USA) using helium as the carrier gas at a constant linear velocity (40 cm/s). The injector temperature was 280 °C, and the detector temperatures were, respectively, 330 °C for FID and 280 °C for the ion source. The targeted fatty acids included markers for Gram-positive bacteria (i15:0, a15:0, i16:0, i17:0, a17:0), Gram-negative bacteria (cy17:0, C18:1ω7, cy19:0), saprotrophs (C18:2w6,9) and arbuscular mycorrhizal fungi (C16:1ω5).

### 4.5. Biological Activity Assessment

#### 4.5.1. Microbial Strains

Four microbiological species, including Gram-positive bacteria (*Staphylococcus aureus* CIP 53154 and *Enterococcus hirae*) and Gram-negative bacteria (*Escherichia coli* CIP 54127 and *Pseudomonas aeruginosa* CIP A22), were used to evaluate *L. dentata*’s EO antimicrobial properties.

#### 4.5.2. Antibacterial Activity

We determined the minimum inhibitory concentration values against the four bacterial strains studied for each EO using broth microdilution in 96-well microplates as described by Güllüce et al. [117]. Test samples were prepared by adding 10 µL of bacterial suspension into each well and incubating with 100 µL of nutrient broth and 100 µL of EO extracts. Subsequently, 100 µL from each dilution was transferred to the next consecutive wells. As a sterility control, 200 µL of the medium supplemented with 10% ethanol was used as an uninoculated medium. Each multiculture plate was sealed and incubated at 37 °C for 24 h. All antibacterial assays were conducted in triplicate (*n* = 3) for each treatment and concentration. Results are reported as the mean ± SD of three independent experiments.

#### 4.5.3. Nematicidal Activity

Nematode inoculum and in vitro nematicidal activity

*Tylenchulus semipenetrans* inoculum:

We collected Citrus plants infested with *Tylenchulus semipenetrans* from the Graoua domain in Marrakech Province, Morocco. Juvenile (J2) nematodes were extracted using the modified Greco and Addabbo [118] method. Roots were washed, dried, cut into 0.5 cm pieces, submerged in 0.5% sodium hypochlorite, blended for three seconds, and washed with sterile water. The suspension was filtered through sieves (100, 60, 45, 30, 20, 5 μm), calibrated to 50 J2s/250 μL, and stored at 4 °C for assays.

*Meloidogyne javanica* inoculum:

The *Meloidogyne javanica* population was originally from naturally infested tomato plants in Belfaa Province, Morocco. Identification was based on adult female perineal patterns [119]. The population was maintained on tomato (*Solanum lycopersicum* L. cv. Twarga). Infected roots were carefully extracted and thoroughly rinsed with running water for inoculum preparation. Egg masses were detached with sterile forceps and sterilized in 0.5% NaOCl for one minute, then washed three times with sterile water [120]. Then, they were incubated in sterile water at 28 °C for four days using the Baermann funnel to obtain second-stage juveniles (J2). The J2 suspension was standardized to 80 juveniles per 200 μL and stored at 4 °C for lab use.

In vitro nematicidal activity assay:

Essential oils (EOs) were prepared using the procedure described by Mc Donnell et al. [121]. The necessary quantities of EOs were mixed with Tween 80 (in a 1:2 ratio, which acts as an emulsifier) and then diluted with sterile distilled water (SDW) to achieve the target concentrations (1, 3, and 5 µL/mL). A 2% Tween 80 solution and sterile DW were utilized as controls [121].

For the juvenile assay, a 1 mL suspension containing 50 second-stage juveniles (J2) of *T. semipenetrans* or *M. javanica* was placed into separate Petri dishes (3 mm in diameter). Next, 1 mL of each EO solution (from either cultivated or wild sources) was added to the dishes containing the nematode suspension. The Petri dishes were incubated at a temperature of 25 ± 2 °C, with the J2 being exposed for 24, 48, and 96 h. Each treatment was replicated five times in a completely randomized design. The mortality of juveniles was assessed using a light microscope.

In vitro anti *Fusarium oxysporum* assay:

*Fusarium oxysporum*, isolated from wilting melon stems, served as the test phytopathogen for this study. The infected stems were cut into small pieces, washed in water, and sterilized with 1% sodium hypochlorite (NaClO). They were subsequently rinsed multiple times with sterilized water to remove any hypochlorite residue. The sterilized stem pieces were transferred to potato dextrose agar (PDA) and incubated at 25 °C for 7 to 10 days. The fungal strain was identified through microscopic analysis of the isolates based on spore structure, size, and colony color using appropriate mycological keys. The antifungal activity against *F. oxysporum* was assessed using the disk microdiffusion method [122]. A sterile Whatman filter paper disc with a 6 mm diameter was placed on top of the solidified PDA medium in each sterile Petri dish. Each disc was filled with 10 µL of the tested EO. To prepare mycelial plugs, 0.5 cm discs of mycelium from a five-day-old culture of *F. oxysporum* were cut and inoculated in the center of the Petri dish on top of the impregnated discs. This step ensured direct contact between the mycelium and the EO being tested. Each treatment was done in triplicate. The plates were incubated for 5 days at 28 °C, after which the percentage of mycelial inhibition was measured following the formula:

Percentage of mycelial inhibition (%) = (D_0_ − D_T_)/D_0_ × 100

where D_0_ is the mean colony diameter for the control sets and D_T_ is the mean colony diameter for the treatment sets.

#### 4.5.4. Antioxidant Activity

DPPH free radical scavenging activity:

The antioxidant activity of the studied EO against DPPH was determined using the method reported by Quiroga et al. [123]. Briefly, a 10 μM solution of DPPH in methanol was prepared, and 900 µL of this solution was added to 100 µL of the tested samples. The mixture was vigorously shaken and incubated in the dark at room temperature for 20 min. The blank used is the ethanolic dilution of DPPH, and the absorbance was therefore determined at 517 nm. The radical scavenging activity was estimated as 50% of the inhibition concentration (IC50) in mg/mL and was calculated as follows:

Measurement of radical scavenging activity:

((Control AB − Sample AB)/Control AB) × 100(1)

#### 4.5.5. ABTS Radical Cation-Scavenging Activity

ABTS radical cation (ABTS) was prepared by mixing a 50:50 *v*/*v* solution of ethanol and water with 2.45 mM potassium persulfate and 7 mM ABTS. This solution was then incubated overnight at 25 °C in the dark. The ABTS+ solution was diluted with ethanol to an absorbance of 0.70 (±0.005) at 734 nm. Subsequently, 2.5 mL of diluted ABTS solution was added to 0.25 mL of EO samples, which were also dissolved in ethanol, and the percentage inhibition of absorbance at 734 nm, expressed as the 50% inhibitory concentration (IC 50) in mg/mL, was calculated using the following formula:

% Inhibition = ((A_control_ − A_sample_)/A_control_) × 100 (2)

Total antioxidant activity (TAC):

Total antioxidant activity (TAC) is the capacity to remove free radicals, as described by Rubio et al. [113,114,115,116,117,118,119,120,121,122,123,124]. There is a reduction of phosphomolybdic acid by sodium sulfide, which forms a blue complex [114,115,116,117,118,119,120,121,122,123,124,125]. First, we mixed 10 mL of sulfuric acid, 49 mg of ammonium molybdate, and 72 mg of sodium phosphate with 10 mL of distilled water. After this process, we added 100 µL of each sample to 900 µL of the regent solution, and we incubated it at 95 °C for 90 min. A volume of 100 µL of distilled water was used instead of the sample for the blank. During the post-incubation period, the solution was allowed to cool down at room temperature, and then the absorbance was measured at 696nm. Results were expressed as the 50% inhibitory concentration (IC50) in mg/mL.

The following equation calculated the total antioxidant activity:

TAC (%) = ((A_sample_ − A_control_)/A_blank_) × 100 (3)

Ferric Reducing/Antioxidant Power (FRAP) Method:

FRAP, or Ferric reducing antioxidant power assay, involving a radical scavenging method, was conducted according to the protocol proposed by Berker et al. [115,116,117,118,119,120,121,122,123,124,125,126]. 200 μL aliquots of diluted EO samples were used at different concentrations, with three replicates for each sample. These aliquots were mixed with 500 µL of phosphate buffer (0.2 M, pH 6.6) and 500 µL of 1% potassium ferricyanide solution. After this, these aliquots were incubated at 50 °C for around 20 min. Post incubation, 500 µL of trichloroacetic acid (10%) was added to each solution, followed by centrifugation at 3000 rpm for 10 min. After centrifugation, 500 μL of distilled water and 100 μL of 0.1% ferric chloride or FeCl_3_ were added and incubated at room temperature for another 10 min. The increase in absorbance was measured at 700 nm. The concentration with 0.5 absorbance is equivalent to a 50% reduction (IC50).

#### 4.5.6. Statistical Analysis

All biological activities data (antibacterial, antifusarium, nematicidal, and antioxidant activities) had been statistically analyzed using RStudio software version 4.4.3. A Student’s *t*-test was applied to compare the means of the mentioned bioactivity parameters measured between cultivated and wild *L. dentata* samples. Pearson correlation heatmaps were generated separately for the cultivated and wild sites to explore the relationships among these variables. It is based on site-specified interactive patterns and ecological dynamics. Only the most significant parameters showing significant variation and biological relevance were included in correlation matrices. Thus, a clear visualization of interdependency among soil characteristics, microbial diversity, and plant biological activities in each ecological context has been achieved.

## 5. Conclusions

This study demonstrated that the microbial abundance in the soil and roots of *L. dentata*, the yield, chemical composition, and biological properties of its EO varied significantly depending on its growth conditions. The microbial abundance in both soil and roots differed between wild and cultivated plants, influenced not only by their nutritional requirements but also by other factors such as soil management practices, organic matter content, microclimatic conditions, and soil physicochemical properties; hence, better results were observed in natural growth conditions. *L. dentata* growth conditions also influenced the chemical profile of the EO between wild and cultivated plants. Several compounds increased or decreased between the two sites, including eucalyptol, borneol, alpha-pinene, and myrtenol. Qualitatively, some compounds characterized wild *L. dentata* EO, such as linalool, cis-verbenol, carveol, and alpha-selinene. In contrast, other compounds were present only in cultivated EO, including tricyclene, d-limonene, camphene hydrate, crypton, and isobornyl acetate. These differences in the chemical profile impacted the biological properties of EO. Our study demonstrates the importance of plant growth conditions in shaping EO chemical composition and yield and affecting their biological activities. These findings highlight that wild populations of *L. dentata* exhibited higher biological activities in some assays; however, the overall differences between wild and cultivated plants were moderate and not consistent across all tests. This underscores the importance of preserving natural ecosystems, while also optimizing agricultural practices to maintain soil health and support the production of cultivated plants with comparable bioactive potential. Together, these strategies help ensure sustainable and reliable sources of medicinal and aromatic plants.

## Figures and Tables

**Table 1:** Chemical composition of the EO (%) of *L. dentata* at the flowering stage of development between wild and cultivated EO. RT—retention time; IK—Retention indices calculated relative to n-alkanes (C-8 to C-40) using capillary column ZB-5MS; IK*—Retention indices found in the literature and The Pherobase; IK**—Retention indices found in NIST database “–” indicates the absence of the compound or that it was not detected in the corresponding sample.

					Area (%)	
Components	RT	IK	IK*	IK**	Wild EO	Cultivated EO
Tricyclene	2.114	927	926	927	–	0.4
α-Pinene	2.173	937	919	938	2.1	5.9
Camphene	2.279	955	964	955	1.2	1.4
β-Pinene	2.444	984	981	980	4.0	2.6
Limonene	2.747	1035	1036	1034	–	2.1
Eucalyptol	2.799	1043	1033	1048	23.1	14.8
γ-Terpinene	2.994	1076	1062	1069	0.5	0.3
Fenchone	3.092	1092	1094	1088	0.4	0.3
Linalool	3.118	1096	1098	1098	0.4	–
β-fenchol	3.17	1105	1117	1112	1.5	0.9
Camphor	3.346	1135	1143	1141	0.5	0.5
Borneol	3.509	1162	1165	1165	49.5	32.9
Camphene hydrate	3.542	1168	1150	1150	–	0.9
Pinocarvone	3.57	1173	1168	1165	1.6	0.9
Crypton	3.624	1182	1188	1183		1.3
Terpinen-4-ol	3.671	1190	1189	1191	3.1	3.8
Myrtenol	3.758	1205	1202	1194	3.7	2.7
Cis-Verbenol	3.833	1218	1216	1214	0.4	–
Carveol	3.894	1229	1229	1229	0.3	–
Isobornyl acetate	4.237	1291	1286	1290	–	24.5
Terpinyl acetate	4.564	1351	1352	1351	–	0.7
β-caryophyllène	5.296	1494	1428	1451	0.3	0.3
β-selinene	5.345	1504	1485	1509	0.8	0.5
α-Selinene	5.434	1522	1494	1505	0.4	–
Caryophyllene oxide	5.803	1599	1581	1596	0.8	0.4
Cubenol	6.024	1646	1642	1644	0.4	0.4
β-Eudesmol	6.136	1670	1654	1653	3.8	1.8
Monoterpene hydrocarbons					7.7	12.5
Oxygenated monoterpenes					84.1	83.7
Hydrocarbon sesquiterpenes					1.5	0.8
Oxygenated sesquiterpenes					4.9	2.6

**Table 2:** Rhizospheric soil’s physicochemical characteristics in cultivated and wild *L. dentata*.

	Wild *L. dentata*	Cultivated *L. dentata*
pH water	7.63 ± 0.13	6.63 ± 0.09
pH KCl Conductivity (µs/cm)	7.27 ± 0.11 107.97 ± 3.21	6.13 ± 0.07 1513 ± 7.41
Organic matter (g/kg)	38.90 ± 3.80	21.67 ± 1.96
Organic carbon (g/kg)	22.60 ± 2.20	12.6 ± 1.14
Nitrogen (g/kg)	3.64 ± 0.35	2.82 ± 0.02
Carbon/Nitrogen	6.20	4.46
Total limestone (g/kg)	10.70 ± 0.99	54.04 ± 1.44
Calcium (g/kg)	11.50 ± 0.37	17.55 ± 0.31
Potassium (g/kg)	0.53 ± 0.06	1.22 ± 0.14
Sodium (g/kg)	0.01 ± 0.008	0.03 ± 0.005
Phosphorus (g/kg)	0.03 ± 0.001	0.08 ± 0.006

## Data Availability

The original contributions presented in this study are included in the article. Further inquiries can be directed to the corresponding authors.

## References

[1] Chaytor D.A. (1937). A taxonomic study of the genus *Lavandula*. Bot. J. Linn. Soc..

[2] Lamrani-Alaoui M., Hassikou R. (2018). Rapid risk assessment to harvesting of wild medicinal and aromatic plant species in Morocco for conservation and sustainable management purposes. Biodivers. Conserv..

[3] González-Coloma A., Delgado F., Rodilla J.M., Silva L., Sanz J., Burillo J. (2011). Chemical and biological profiles of *Lavandula luisieri* essential oils from western Iberia Peninsula populations. Biochem. Syst. Ecol..

[4] Touati B., Chograni H., Hassen I., Boussaïd M., Toumi L., Brahim N.B. (2011). Chemical composition of the leaf and flower essential oils of Tunisian *Lavandula dentata* L.(Lamiaceae). Chem. Biodivers..

[5] Gallotte P., Fremondière G., Gallois P., Bernier J.-P.B., Buchwalder A., Walton A., Piasentin J., Fopa-Fomeju B. (2020). Lavandula angustifolia mill. And *Lavandula* x intermedia emeric ex loisel: Lavender and lavandin. Medicinal, Aromatic and Stimulant Plants.

[6] Rezaei M.N., Dornez E., Jacobs P., Parsi A., Verstrepen K.J., Courtin C.M. (2014). Harvesting yeast (*Saccharomyces cerevisiae*) at different physiological phases significantly affects its functionality in bread dough fermentation. Food Microbiol..

[7] Algieri F., Rodriguez-Nogales A., Vezza T., Garrido-Mesa J., Garrido-Mesa N., Utrilla M.P., González-Tejero M.R., Casares-Porcel M., Molero-Mesa J., del Mar Contreras M. (2016). Anti-inflammatory activity of hydroalcoholic extracts of *Lavandula dentata* L. and *Lavandula stoechas* L.. J. Ethnopharmacol..

[8] Wagner L.S., Sequin C.J., Foti N., Campos-Soldini M.P. (2021). Insecticidal, fungicidal, phytotoxic activity and chemical composition of *Lavandula dentata* essential oil. Biocatal. Agric. Biotechnol..

[9] Zuzarte M., Vale-Silva L., Gonçalves M.J., Cavaleiro C., Vaz S., Canhoto J., Pinto E., Salgueiro L. (2012). Antifungal activity of phenolic-rich *Lavandula multifida* L. essential oil. Eur. J. Clin. Microbiol. Infect. Dis..

[10] Toda M., Matsuse R. (2020). Endocrinological effect of Lavender aromatherapy on stressful visual stimuli. Contemp. Clin. Trials Commun..

[11] Zhang S., Zhang L., Zou H., Qiu L., Zheng Y., Yang D., Wang Y. (2021). Effects of light on secondary metabolite biosynthesis in medicinal plants. Front. Plant Sci..

[12] Selwal N., Rahayu F., Herwati A., Latifah E., Supriyono, Suhara C., Suastika I.B.K., Mahayu W.M., Wani A.K. (2023). Enhancing secondary metabolite production in plants: Exploring traditional and modern strategies. J. Agric. Food Res..

[13] Akachoud O., Bouamama H., Laruelle F., Facon N., Broudi S.E.L., Houssayni S., Zoubi B., Benkebboura A., Ghoulam C., Sahraoui A.L.-H. (2024). The developmental stage and arbuscular mycorrhizal symbiosis influence the essential oil yield, chemical profile, and biological activities in *Thymus pallidus*, *T. satureioides*, and *Lavandula dentata*. Ind. Crops Prod..

[14] Hamilton A.C. (2004). Medicinal plants, conservation and livelihoods. Biodivers. Conserv..

[15] Abbad A., Belaqziz R., Bekkouche K., Markouk M. (2011). Influence of temperature and water potential on laboratory germination of two Moroccan endemic thymes: *Thymus maroccanus Ball.* and Thymus broussonetii Boiss. Afr. J. Agric. Res..

[16] El-Bakkal S.E., Zeroual S., Elouazkiti M., Mansori M., Bouamama H., Zehhar N., El-Kaoua M. (2020). Comparison of yield chemical composition and biological activities of essential oils obtained from *Thymus pallidus* and *Thymus satureioides* Coss. grown in wild and cultivated conditions in Morocco. J. Essent. Oil Bear. Plants.

[17] Lubbe A., Verpoorte R. (2011). Cultivation of medicinal and aromatic plants for specialty industrial materials. Ind. Crops Prod..

[18] Carrión V.J., Perez-Jaramillo J., Cordovez V., Tracanna V., de Hollander M., Ruiz-Buck D., Mendes L.W., van Ijcken W.F.J., Gomez-Exposito R., Elsayed S.S. (2019). Pathogen-induced activation of disease-suppressive functions in the endophytic root microbiome. Science.

[19] De Vries F.T., Griffiths R.I., Knight C.G., Nicolitch O., Williams A. (2020). Harnessing rhizosphere microbiomes for drought-resilient crop production. Science.

[20] Liu H., Brettell L.E., Qiu Z., Singh B.K. (2020). Microbiome-mediated stress resistance in plants. Trends Plant Sci..

[21] Zhang S., Li M., Cui X., Pan Y. (2023). Effect of different straw retention techniques on soil microbial community structure in wheat–maize rotation system. Front. Microbiol..

[22] Akachoud O., Bouamama H., Facon N., Laruelle F., Zoubi B., Benkebboura A., Ghoulam C., Qaddoury A., Lounès-Hadj Sahraoui A. (2022). Mycorrhizal inoculation improves the quality and productivity of essential oil distilled from three aromatic and medicinal plants: *Thymus satureioides*, *Thymus pallidus*, and *Lavandula dentata*. Agronomy.

[23] Khmelevtsova L.E., Sazykin I.S., Azhogina T.N., Sazykina M.A. (2022). Influence of agricultural practices on bacterial community of cultivated soils. Agriculture.

[24] Pandey A., Singh S. (2016). Aloe vera: A systematic review of its industrial and ethno-medicinal efficacy. Int. J. Pharm. Res. Allied Sci..

[25] Lerotholi L., Chaudhary S.K., Combrinck S., Viljoen A. (2017). Bush tea (*Athrixia phylicoides*): A review of the traditional uses, bioactivity and phytochemistry. S. Afr. J. Bot..

[26] Thakur M., Bhattacharya S., Khosla P.K., Puri S. (2019). Improving production of plant secondary metabolites through biotic and abiotic elicitation. J. Appl. Res. Med. Aromat. Plants.

[27] Martins R.D.P., Gomes R.A.D.S., Malpass A.C.G., Okura M.H. (2019). Chemical characterization of *Lavandula dentata* L. essential oils grown in Uberaba-MG. Ciênc. Rural.

[28] El Abdali Y., Agour A., Allali A., Bourhia M., El Moussaoui A., Eloutassi N., Salamatullah A.M., Alzahrani A., Ouahmane L., Aboul-Soud M.A.M. (2022). *Lavandula dentata* L.: Phytochemical analysis, antioxidant, antifungal and insecticidal activities of its essential oil. Plants.

[29] Belcadi H., Aknouch A., Chraka A., Kassout J., Lachkar M., Mouhib M., Mansour A.I. (2024). Moroccan *Lavandula dentata* L. essential oil: γ-Irradiation effect on the chemical composition and antibacterial activity. Sci. Afr..

[30] Sile I., Krizhanovska V., Nakurte I., Mezaka I., Kalane L., Filipovs J., Vecvanags A., Pugovics O., Grinberga S., Dambrova M. (2022). Wild-grown and cultivated *Glechoma hederacea* L.: Chemical composition and potential for cultivation in organic farming conditions. Plants.

[31] Abdellaoui M., Derouich M., El-Rhaffari L. (2020). Essential oil and chemical composition of wild and cultivated fennel (*Foeniculum vulgare Mill*.): A comparative study. S. Afr. J. Bot..

[32] Atyane L.H., Lagram K., Ben El Caid M., Lachheb M., Salaka L., Serghini M.A., Elmaimouni L. Study of the influence of geographical origin and environment conditions on the three secondary metabolites of Moroccan saffron by UV-visible spectrometry. Proceedings of the V International Symposium on Saffron Biology and Technology: Advances in Biology, Technologies, Uses and Market.

[33] Kumar S., Saini R., Suthar P., Kumar V., Sharma R. (2022). Plant secondary metabolites: Their food and therapeutic importance. Plant Secondary Metabolites: Physico-Chemical Properties and Therapeutic Applications.

[34] Chrysargyris A., Tzortzakis N. (2025). Nitrogen, Phosphorus, and Potassium Requirements to Improve *Portulaca oleracea* L. Growth, Nutrient and Water Use Efficiency in Hydroponics. Agronomy.

[35] Villette J., Cuéllar T., Verdeil J.L., Delrot S., Gaillard I. (2020). Grapevine potassium nutrition and fruit quality in the context of climate change. Front. Plant Sci..

[36] Willy D.K., Muyanga M., Mbuvi J., Jayne T. (2019). The effect of land use change on soil fertility parameters in densely populated areas of Kenya. Geoderma.

[37] Niinemets Ü., Seufert G., Steinbrecher R., Tenhunen J.D. (2002). A model coupling foliar monoterpene emissions to leaf photosynthetic characteristics in Mediterranean evergreen *Quercus* species. New Phytol..

[38] Erbaş S., Kucukyumuk Z., Baydar H., Erdal İ., Sanlı A. (2017). Effects of different phosphorus doses on nutrient concentrations as well as yield and quality characteristics of lavandin (*Lavandula* × *intermedia* Emeric ex Loisel. var. Super). Turk. J. Field Crops.

[39] Amanuel W., Yimer F., Karltun E. (2018). Soil organic carbon variation in relation to land use changes: The case of Birr watershed, upper Blue Nile River Basin, Ethiopia. J. Ecol. Environ..

[40] Murindangabo Y.T., Kopecký M., Hoang T.N., Bernas J., Parajuli T., Dhakal S., Konvalina P., de Dieu Marcel Ufitikirezi J., Kaneza G., Khanal B.R. (2023). Comparative analysis of soil organic matter fractions, lability, stability ratios, and carbon management index in various land use types within bharatpur catchment, Chitwan District, Nepal. Carbon Balance Manag..

[41] Gerke J. (2022). The central role of soil organic matter in soil fertility and carbon storage. Soil Syst..

[42] Hoffland E., Kuyper T.W., Comans R.N., Creamer R.E. (2020). Eco-functionality of organic matter in soils. Plant Soil..

[43] Chaouqi S., Moratalla-López N., Alonso G.L., Lorenzo C., Zouahri A., Asserar N., Haidar E.M., Guedira T. (2023). Effect of soil composition on secondary metabolites of moroccan saffron (*Crocus sativus* L.). Plants.

[44] Blanch J.S., Peñuelas J., Llusià J. (2007). Sensitivity of terpene emissions to drought and fertilization in terpene-storing *Pinus halepensis* and non-storing *Quercus ilex*. Physiol. Plant..

[45] Ormeño E., Goldstein A., Niinemets Ü. (2011). Extracting and trapping biogenic volatile organic compounds stored in plant species. TrAC Trends Anal. Chem..

[46] Koeduka T., Fridman E., Gang D.R., Vassão D.G., Jackson B.L., Kish C.M., Orlova I., Spassova S.M., Lewis N.G., Noel J.P. (2006). Eugenol and isoeugenol, characteristic aromatic constituents of spices, are biosynthesized via reduction of a coniferyl alcohol ester. Proc. Natl. Acad. Sci. USA.

[47] Hazrati S., Mousavi Z., Mollaei S., Sedaghat M., Mohammadi M., Pignata G., Nicola S. (2024). Optimizing Nitrogen Fertilization to Maximize Yield and Bioactive Compounds in *Ziziphora clinopodioides*. Agriculture.

[48] Santoyo G., Urtis-Flores C.A., Loeza-Lara P.D., Orozco-Mosqueda M.D.C., Glick B.R. (2021). *Rhizosphere* colonization determinants by plant growth-promoting rhizobacteria (PGPR). Biology.

[49] Sasse J., Martinoia E., Northen T. (2018). Feed your friends: Do plant exudates shape the root microbiome. Trends Plant Sci..

[50] Olanrewaju O.S., Ayangbenro A.S., Glick B.R., Babalola O.O. (2019). Plant health: Feedback effect of root exudates-rhizobiome interactions. Appl. Microbiol. Biotechnol..

[51] Andreote F.D., Gumiere T., Durrer A. (2014). Exploring interactions of plant microbiomes. Sci. Agríc..

[52] Potthoff M., Steenwerth K.L., Jackson L.E., Drenovsky R.E., Scow K.M., Joergensen R.G. (2006). Soil microbial community composition as affected by restoration practices in California grassland. Soil Biol. Biochem..

[53] Yang W., Yan Y., Jiang F., Leng X., Cheng X., An S. (2016). Response of the soil microbial community composition and biomass to a short-term *Spartina alterniflora* invasion in a coastal wetland of eastern China. Plant Soil.

[54] Manral V., Bargali K., Bargali S.S., Shahi C. (2020). Changes in soil biochemical properties following replacement of Banj oak forest with Chir pine in Central Himalaya, India. Ecol. Process..

[55] Shao W., Li M., Wu Y., Ma X., Song Q., Zhang Y., Su Y., Ni J., Dong J. (2022). Identification of varied soil hydraulic properties in a seasonal tropical rainforest. Catena.

[56] Chiba A., Uchida Y., Kublik S., Vestergaard G., Buegger F., Schloter M., Schulz S. (2021). Soil bacterial diversity is positively correlated with decomposition rates during early phases of maize litter decomposition. Microorganisms.

[57] Zhou J., Guan D., Zhou B., Zhao B., Ma M., Qin J., Jiang X., Chen S., Cao F., Shen D. (2015). Influence of 34-years of fertilization on bacterial communities in an intensively cultivated black soil in northeast China. Soil Biol. Biochem..

[58] Huang Q., Wang J., Wang C., Wang Q. (2019). The 19-years inorganic fertilization increased bacterial diversity and altered bacterial community composition and potential functions in a paddy soil. Appl. Soil Ecol..

[59] Rousk J., Bååth E., Brookes P.C., Lauber C.L., Lozupone C., Caporaso J.G., Knight R., Fierer N. (2010). Soil bacterial and fungal communities across a pH gradient in an arable soil. ISME J..

[60] Wang N., Zhang T., Li Y., Cong A., Lian J., Feng K. (2025). Integrated application of fertilization increased maize (*Zea mays* L.) yield by improving soil quality, particularly under limited water conditions in a semi-arid sandy area. Agric. Water Manag..

[61] Fang X., Wang M., Zhou X., Wang H., Wang H., Xiao H. (2022). Effects of growth years on ginsenoside biosynthesis of wild ginseng and cultivated ginseng. BMC Genom..

[62] Kokkoris V., Hamel C., Hart M.M. (2019). Mycorrhizal response in crop versus wild plants. PLoS ONE.

[63] Jackson L.E., Miller D., Smith S.E. (2002). Arbuscular mycorrhizal colonization and growth of wild and cultivated lettuce in response to nitrogen and phosphorus. Sci. Hortic..

[64] Rillig M.C., Sosa-Hernández M.A., Roy J., Aguilar-Trigueros C.A., Vályi K., Lehmann A. (2016). Towards an integrated mycorrhizal technology: Harnessing mycorrhiza for sustainable intensification in agriculture. Front. Plant Sci..

[65] Hontoria C., García-González I., Quemada M., Roldán A., Alguacil M.M. (2019). The cover crop determines the AMF community composition in soil and in roots of maize after a ten-year continuous crop rotation. Sci. Total Environ..

[66] Strom N., Hu W., Haarith D., Chen S., Bushley K. (2020). Interactions between soil properties, fungal communities, the soybean cyst nematode, and crop yield under continuous corn and soybean monoculture. Appl. Soil Ecol..

[67] Yuan H., Ma Q., Ye L., Piao G. (2016). The traditional medicine and modern medicine from natural products. Molecules.

[68] Zhai X., Jia M., Chen L., Zheng C.J., Rahman K., Han T., Qin L.P. (2017). The regulatory mechanism of fungal elicitor-induced secondary metabolite biosynthesis in medical plants. Crit. Rev. Microbiol..

[69] Zhou J.Y., Li X., Zheng J.Y., Dai C.C. (2016). Volatiles released by endophytic *Pseudomonas fluorescens* promoting the growth and volatile oil accumulation in *Atractylodes lancea*. Plant Physiol. Biochem..

[70] Zheng L.P., Tian H., Yuan Y.F., Wang J.W. (2016). The influence of endophytic *Penicillium oxalicum* B4 on growth and artemisinin biosynthesis of in vitro propagated plantlets of *Artemisia annua* L.. Plant Growth Regul..

[71] Wang X.M., Yang B., Ren C.G., Wang H.W., Wang J.Y., Dai C.C. (2015). Involvement of abscisic acid and salicylic acid in signal cascade regulating bacterial endophyte-induced volatile oil biosynthesis in plantlets of *Atractylodes lancea*. Physiol. Plant..

[72] Chen L., Liu Y. (2024). The function of root exudates in the root colonization by beneficial soil rhizobacteria. Biology.

[73] Kashid S., Joshi K., More S., Shinde A., Nene S. (2023). Enhanced Productivity of Fragrance Compounds: Biotransformation of d-limonene Using Whole Cell Immobilization of *Pseudomonas putida* and *Rhodococcus erythropolis*. J. Inst. Eng. India Ser. E.

[74] Epand R.M., Walker C., Epand R.F., Magarvey N.A. (2016). Molecular mechanisms of membrane targeting antibiotics. Biochim. Biophys. Acta Biomembr..

[75] Gachkar L., Yadegari D., Rezaei M.B., Taghizadeh M., Astaneh S.A., Rasooli I. (2007). Chemical and biological characteristics of *Cuminum cyminum* and *Rosmarinus officinalis* essential oils. Food Chem..

[76] Su J., Chen J., Liao S., Li L., Zhu L., Chen L. (2012). Composition and biological activities of the essential oil extracted from a novel plant of *Cinnamomum camphora Chvar.*. Borneol. J. Med. Plants Res..

[77] Leite-Sampaio N.F., Gondim C.N.F., de Souza C.E.S., Coutinho H.D. (2022). Antibiotic potentiating action of α-PINENE and borneol against EPEC and ETEC sorotypes. Microb. Pathog..

[78] Yu H., Ren X., Yang F., Xie Y., Guo Y., Cheng Y., Yao W. (2022). Antimicrobial and anti-dust mite efficacy of *Cinnamomum camphora chvar. Borneol* essential oil using pilot-plant neutral cellulase-assisted steam distillation. Lett. Appl. Microbiol..

[79] Ma R., Lu D., Wang J., Xie Q., Guo J. (2023). Comparison of pharmacological activity and safety of different stereochemical configurations of borneol: L-borneol, D-borneol, and synthetic borneol. Biomed. Pharmacother..

[80] Soković M., Glamočlija J., Marin P.D., Brkić D., Van Griensven L.J. (2010). Antibacterial effects of the essential oils of commonly consumed medicinal herbs using an in vitro model. Molecules.

[81] Rosato A., Vitali C., De Laurentis N., Armenise D., Milillo M.A. (2007). Antibacterial effect of some essential oils administered alone or in combination with Norfloxacin. Phytomedicine.

[82] Farhanghi A., Aliakbarlu J., Tajik H., Mortazavi N., Manafi L., Jalilzadeh-Amin G. (2022). Antibacterial interactions of pulegone and 1, 8-cineole with monolaurin ornisin against *Staphylococcus aureus*. Food Sci. Nutr..

[83] Sun Y., Cai X., Cao J., Wu Z., Pan D. (2018). Effects of 1,8-cineole on carbohydrate metabolism related cell structure changes of *Salmonella*. Front. Microbiol..

[84] Chen J., Tang C., Zhou Y., Zhang R., Ye S., Zhao Z., Lin L., Yang D. (2020). Anti-inflammatory property of the essential oil from *Cinnamomum camphora* (Linn.) Presl leaves and the evaluation of its underlying mechanism by using metabolomics analysis. Molecules.

[85] Patterson A.D., Carlson B.A., Li F., Bonzo J.A., Yoo M.H., Krausz K.W., Conrad M., Chen C., Gonzalez F.J., Hatfield D.L. (2013). Disruption of thioredoxin reductase 1 protects mice from acute acetaminophen-induced hepatotoxicity through enhanced NRF2 activity. Chem. Res. Toxicol..

[86] Hachlafi N.E., Aanniz T., Menyiy N.E., Baaboua A.E., Omari N.E., Balahbib A., Shariati M.A., Zengin G., Fikri-Benbrahim K., Bouyahya A. (2023). In vitro and in vivo biological investigations of camphene and its mechanism insights: A review. Food Rev. Int..

[87] Ruberto G., Baratta M.T. (2000). Antioxidant activity of selected essential oil components in two lipid model systems. Food Chem..

[88] Badawy M.E., Marei G.I.K., Rabea E.I., Taktak N.E. (2019). Antimicrobial and antioxidant activities of hydrocarbon and oxygenated monoterpenes against some foodborne pathogens through in vitro and in silico studies. Pestic. Biochem. Physiol..

[89] Zhang Y., Tian Z., Huang T., Lei L., Zuo Z. (2025). Sesquiterpene emissions from four chemotypes of *Cinnamomum camphora* in different seasons. Ind. Crops Prod..

[90] Ortiz de Elguea-Culebras G., Sánchez-Vioque R., Berruga M.I., Herraiz-Peñalver D., González-Coloma A., Andrés M.F., Santana-Méridas O. (2018). Biocidal potential and chemical composition of industrial essential oils from *Hyssopus officinalis*, *Lavandula*× intermedia var. super, and *Santolina chamaecyparissus*. Chem. Biodivers..

[91] D’Addabbo T., Laquale S., Argentieri M.P., Bellardi M.G., Avato P. (2021). Nematicidal activity of essential oil from lavandin (*Lavandula*× intermedia Emeric ex Loisel.) as related to chemical profile. Molecules.

[92] Uludamar E.B.K. (2023). Screening of the nematicidal potential of some essential oils against the Columbia root-knot nematode, *Meloidogyne chitwoodi*. Çukurova Tarım Gıda Bilim. Derg..

[93] Sarri K., Mourouzidou S., Ntalli N., Monokrousos N. (2024). Recent advances and developments in the nematicidal activity of essential oils and their components against root-knot nematodes. Agronomy.

[94] Dutta A., Mandal A., Kundu A., Malik M., Chaudhary A., Khan M.R., Shanmugam V., Rao U., Saha S., Patanjali N. (2021). Deciphering the behavioral response of *Meloidogyne incognita* and *Fusarium oxysporum* toward mustard essential oil. Front. Plant Sci..

[95] Padilla-Montaño N., de León Guerra L., Moujir L. (2021). Antimicrobial activity and mode of action of celastrol, a nortriterpen quinone isolated from natural sources. Foods.

[96] Caballero-Gallardo K., Olivero-Verbel J., Nayive P.B., Stashenko E.E. (2014). Chemical composition and bioactivity of *Piper auritum* and *P. multiplinervium* essential oils against the red flour beetle, *Tribolium castaneum* (Herbst). Bol. Latinoam. Caribe Plantas Med. Aromát..

[97] Zore G.B., Thakre A.D., Jadhav S., Karuppayil S.M. (2011). Terpenoids inhibit *Candida albicans* growth by affecting membrane integrity and arrest of cell cycle. Phytomedicine.

[98] de Macêdo Andrade A.C., Rosalen P.L., Freires I.A., Scotti L., Scotti M.T., Aquino S.G., de Castro R.D. (2018). Antifungal activity, mode of action, docking prediction and anti-biofilm effects of (+)-β-pinene enantiomers against *Candida* spp.. Curr. Top. Med. Chem..

[99] Kubicek C.P., Druzhinina I.S. (2007). Environmental and Microbial Relationships.

[100] Mastouri F., Björkman T., Harman G.E. (2012). *Trichoderma harzianum* enhances antioxidant defense of tomato seedlings and resistance to water deficit. Mol. Plant-Microbe Interact..

[101] Rais A., Jabeen Z., Shair F., Hafeez F.Y., Hassan M.N. (2017). *Bacillus* spp., a bio-control agent enhances the activity of antioxidant defense enzymes in rice against *Pyricularia oryzae*. PLoS ONE.

[102] Padró M.D.A., Caboni E., Morin K.A.S., Mercado M.A.M., Olalde-Portugal V. (2021). Effect of *Bacillus subtilis* on antioxidant enzyme activities in tomato grafting. PeerJ.

[103] Ding X., Jiang Y., Zhao H., Guo D., He L., Liu F., Zhou Q., Nandwani D., Hui D., Yu J. (2018). Electrical conductivity of nutrient solution influenced. photosynthesis, quality, and antioxidant enzyme activity of pakchoi (*Brassica campestris* L. ssp. Chinensis) in a hydroponicsystem. PLoS ONE.

[104] Patra N., Hariharan S., Gain H., Maiti M.K., Das A., Banerjee J. (2021). TypiCal but DeliCate Ca++ re: Dissecting the essence of calcium signaling network as a robust response coordinator of versatile abiotic and biotic stimuli in plants. Front. Plant Sci..

[105] Sikder M.M., Vestergård M. (2020). Impacts of root metabolites on soil nematodes. Front. Plant Sci..

[106] Abdel-Fattah G.M., El-Haddad S.A., Hafez E.E., Rashad Y.M. (2011). Induction of defense responses in common bean plants by arbuscular mycorrhizal fungi. Microbiol. Res..

[107] Boyno G., Rezaee Danesh Y., Çevik R., Teniz N., Demir S., Durak E.D., Farda B., Mignini A., Djebaili R., Pellegrini M. (2025). Synergistic benefits of AMF: Development of sustainable plant defense system. Front. Microbiol..

[108] Barros F.M.D.R., Pedrinho A., Mendes L.W., Freitas C.C.G., Andreote F.D. (2022). Interactions between soil bacterial diversity and plant-parasitic nematodes in soybean plants. Appl. Environ. Microbiol..

[109] Zhang S., Hu W., Zhang J., Yu G., Liu Y., Kong Z., Wu L. (2024). Long-term cultivation reduces soil carbon storage by altering microbial network complexity and metabolism activity in macroaggregates. Sci. Total Environ..

[110] Chen Q., Song Y., An Y., Lu Y., Zhong G. (2024). Mechanisms and Impact of rhizosphere microbial metabolites on crop health, traits, functional components: A comprehensive review. Molecules.

[111] Richards L.A. (1954). Diagnosis and Improvement of Saline and Alkali Soils (No. 60).

[112] Aubert G. (1978). Méthodes D’analyse des Sols Edition.

[113] Barbano D.M., Clark J.L., Dunham C.E., Flemin R.J. (1990). Kjeldahl method for determination of total nitrogen content of milk: Collaborative study. J. Assoc. Off. Anal. Chem..

[114] Olsen S.R., Watanabe F.S., Bowman R.A. (1983). Evaluation of fertilizer phosphate residues by plant uptake and extractable phosphorus. Soil Sci. Soc. Am. J..

[115] Michel-Dewez N., Ek C. (1982). Méthode rapide de caractérisation des dolomies et calcaires magnésiens: La gaz-volumétrie. Bull. Soc. Géogr. Liège.

[116] Brown J.G., Lilleland O. (1946). Rapid determination of potassium and sodium in plant materials and soil extracts by flame photometry. Proc. Am. Soc. Hortic. Sci..

[117] Güllüce M., Sökmen M., Daferera D., Ağar G., Özkan H., Kartal N., Polissiou M., Sökmen A., Şahin F. (2003). In vitro antibacterial, antifungal, and antioxidant activities of the essential oil and methanol extracts of herbal parts and callus cultures of *Satureja hortensis* L.. J. Agric. Food Chem..

[118] Greco N., D’Addabbo T. (1990). Efficient procedure for extracting *Tylenchulus semipenetrans* from citrus roots. J. Nematol..

[119] Taylor A.L., Sasser J.N. (1978). Biology, Identification and Control of Root-Knot Nematodes.

[120] Yang Y.M., Liu P., Dong H., Zhang W.T., Hu X.Q. (2020). Pathogen identification of *Eupatorium adenophorum* root-knot nematode disease in Yunnan Province. J. Plant Prot..

[121] Mc Donnell R., Yoo J., Patel K., Rios L., Hollingsworth R., Millar J., Paine T. (2016). Can essential oils be used as novel drench treatments for the eggs and juveniles of the pest snail *Cornu aspersum* in potted plants?. J. Pest Sci..

[122] Zoubi B., Mokrini F., Amer M., Cherki G., Rafya M., Benkebboura A., Akachoudc O., Laasli S.-E., Housseini A.I., Dababat A.A. (2023). Eco-friendly management of the citrus nematode *Tylenchulus semipenetrans* using some aromatic and medicinal plants. Arch. Phytopathol. Plant Prot..

[123] Quiroga P.R., Nepote V., Baumgartner M.T. (2019). Contribution of organic acids to α-terpinene antioxidant activity. Food Chem..

[124] Rubio C.P., Hernández-Ruiz J., Martinez-Subiela S., Tvarijonaviciute A., Ceron J.J. (2016). Spectrophotometric assays for total antioxidant capacity (TAC) in dog serum: An update. BMC Vet. Res..

[125] Zatar N.A., Abu-Eid M.A., Eid A.F. (1999). Spectrophotometric determination of nitrite and nitrate using phosphomolybdenum blue complex. Talanta.

[126] Berker K.I., Güçlü K., Tor İ., Apak R. (2007). Comparative evaluation of Fe (III) reducing power-based antioxidant capacity assays in the presence of phenanthroline, batho-phenanthroline, tripyridyltriazine (FRAP), and ferricyanide reagents. Talanta.

